# Supplementary material for: Interleukin-10-Mediated Lymphopenia Caused by Acute Infection with Foot-and-Mouth Disease Virus in Mice
Source: Viruses. 2021 Nov 24;13(12):2358. doi: 10.3390/v13122358 (PMC8708299; doi:10.3390/v13122358)
Supplement: Supplementary file 1 [file viruses-13-02358-s001.zip › viruses-1411797-supplementary.pdf]

Table S1. The detailed information of antibodies used for flow cytometry.

Antibody	Fluorescence	Clone	Company	Catalog
Anti-mouse CD3	APC	K3T	BD	MCA500APC
Anti-mouse CD3	PE	145-2c11	Biolegend	100307
Anti-mouse CD3	PE/Cy7	145-2c11	Biolegend	100302
Anti-mouse CD19	PE	6D5	Biolegend	115508
Anti-mouse CD19	APC	6D5	Biolegend	115512
Anti-mouse NK1.1	APC	PK136	Biolegend	108710
Anti-mouse NK1.1	PE	PK136	Biolegend	108708
Anti-mouse CD4	FITC	GK1.5	BD	MCA4635F
Anti-mouse CD8	PE	YTS169.4	BD	MCA1768PE
Anti-mouse CD8	Percp	53-6.7	Biolegend	100732
Anti-mouse CD244 (2B4)	APC	m2B4 (B6)458.1	Biolegend	133517
APC isotype control	APC	MOPC-21	Biolegend	400119
Anti-mouse CD160	PE	7H1	Biolegend	143003
PE isotype control	PE	RTK2758	Biolegend	400507
Anti-mouse CD223 (LAG-3)	APC	C9B7W	Biolegend	125210
APC isotype control	APC	RTK2071	Biolegend	400411
Anti-mouse CD366 (Tim-3)	PE/Cy7	B8.2C12	Biolegend	134010
PE/Cy7 isotype control	PE/Cy7	RTK2071	Biolegend	400415
Anti-mouse TIGIT	BV421	1G9	Biolegend	142111
BV421 isotype control	BV421	MOPC-21	Biolegend	400157
Anti-mouse PD-1	PE/Cy7	29F.1A12	Biolegend	135216
PE/Cy7 isotype control	PE/Cy7	RTK2758	Biolegend	400521
Anti-mouse CTLA-4	PE	UC10-4B9	Biolegend	106305
CTLA-4 isotype control	PE	HTK888	Biolegend	400907

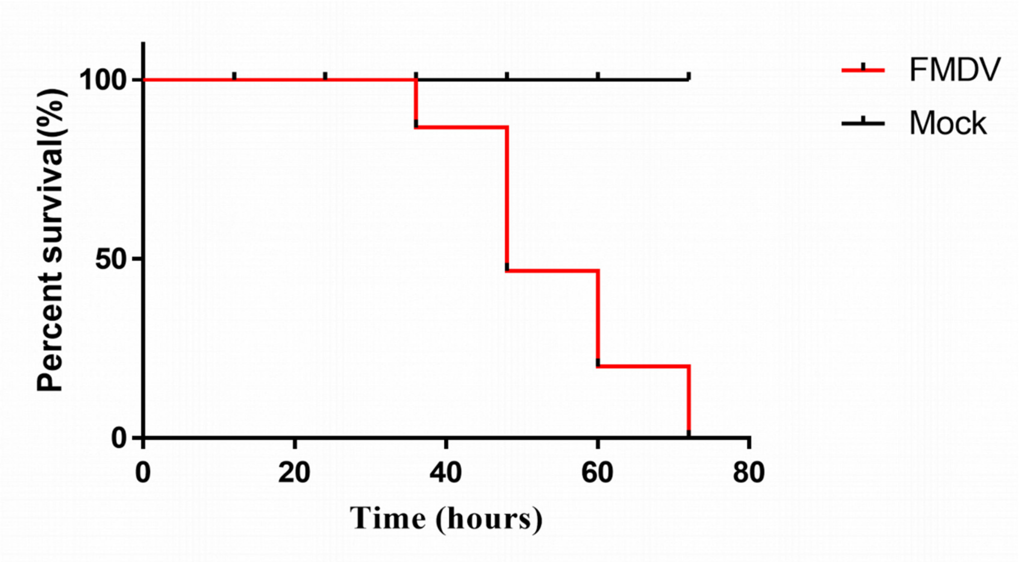

Figure S1. The survival curve of mice infected with FMDV (n=15).

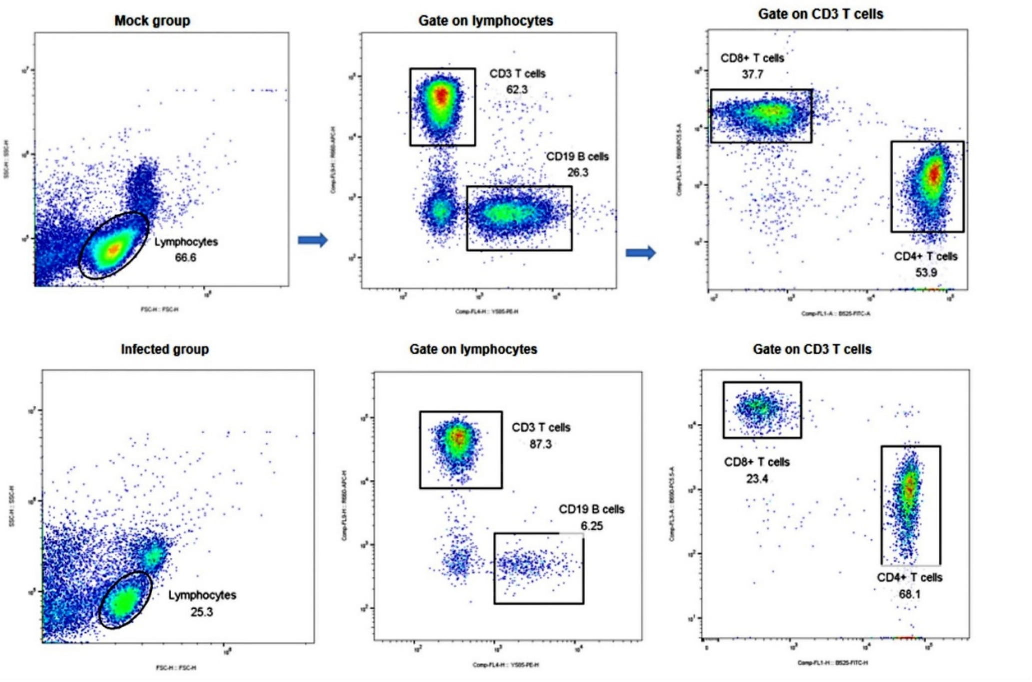

Figure S2. Representative FACS plots of CD3+T cells, CD19+B cells, CD3+CD4+ and CD3+CD8+ T cells from mock and FMDV-infected mice at 48 hpi.

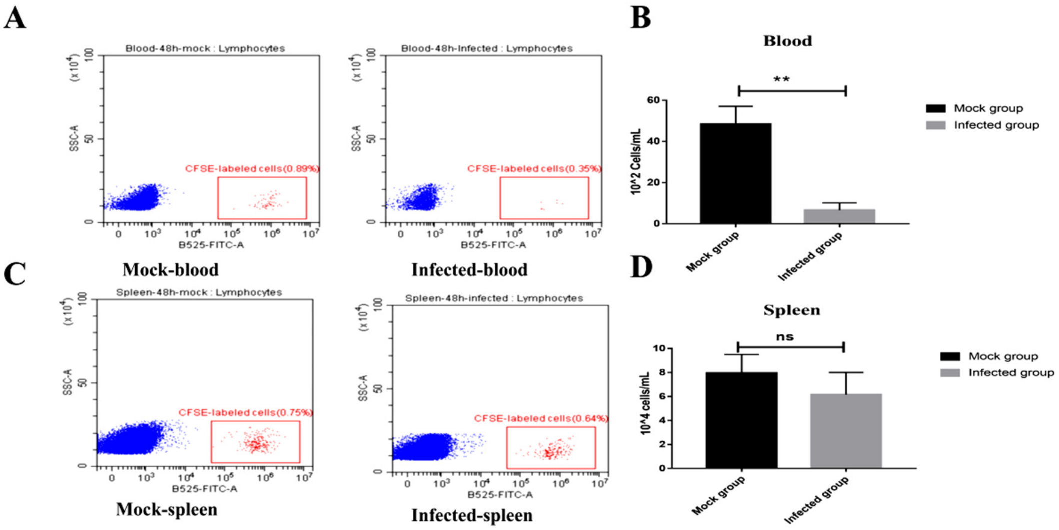

Figure S3. The CFSE-labeled lymphocytes in peripheral blood and spleen. A). Representative FACS plots of CFSE-labeled lymphocytes in blood from mock and FMDV-infected mice. B). CFSE-labeled lymphocytes in blood were significantly decreased in FMDV-infected mice compared with mock mice. C). Representative FACS plots of CFSE-labeled lymphocytes in spleen from mock and FMDV-infected mice. D). CFSE-labeled lymphocytes in spleen were decreased in FMDV-infected mice compared with mock mice.

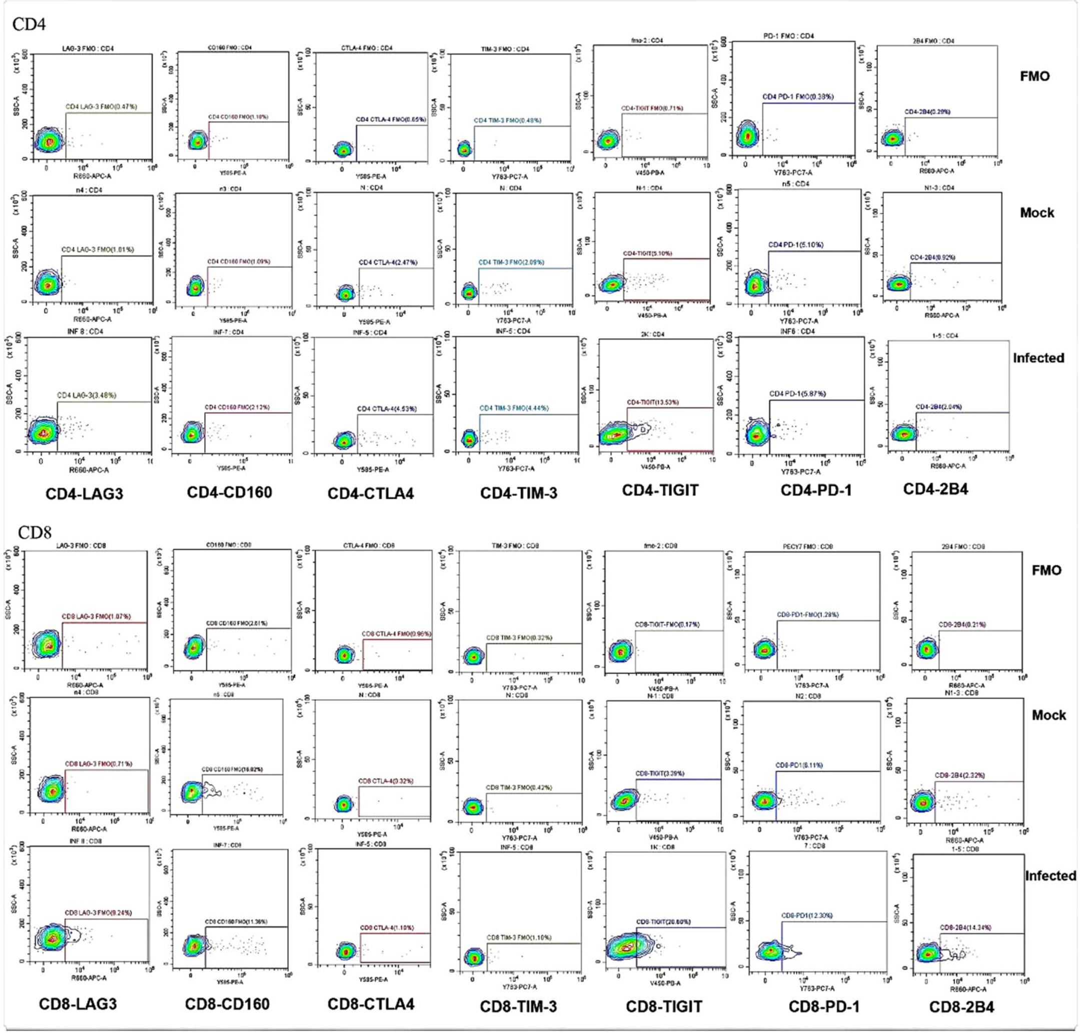

Figure S4. Representative FACS plots of PD-1, CTLA-4, Tim-3, LAG-3, 2B4, TIGIT and CD160 on CD4 and CD8 T cells from mock and FMDV-infected mice.

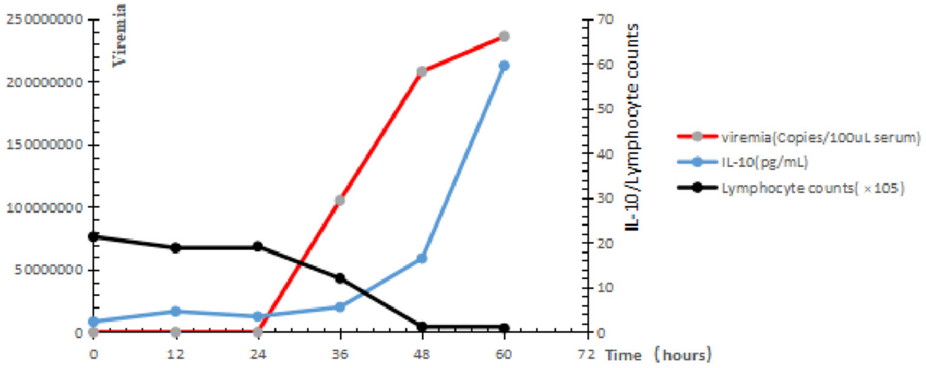

Figure S5. The trend of viremia, lymphocytes count in peripheral blood, and IL-10 concentration in serum during FMDV infection.

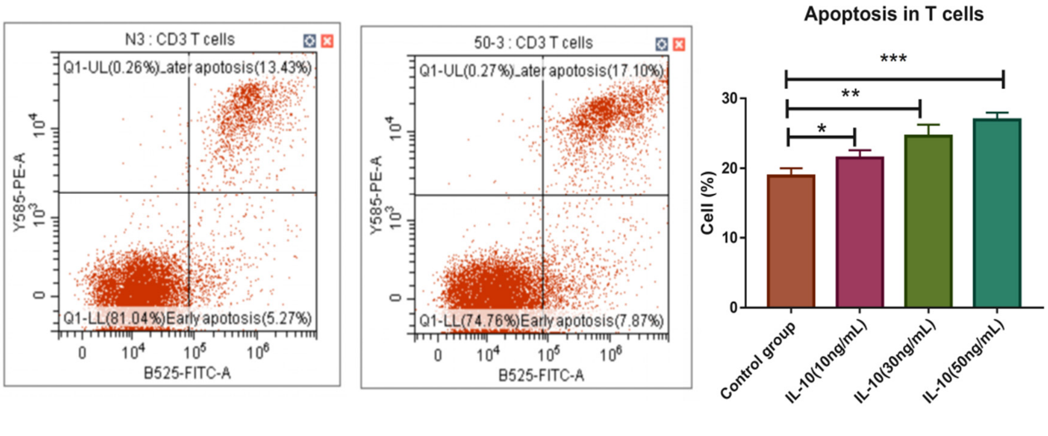

Figure S6. T cells apoptosis was induced by IL-10 *in vitro*.
